# Magnesium in CKD: more than a calcification inhibitor?

**DOI:** 10.1007/s40620-014-0140-6

**Published:** 2014-09-17

**Authors:** Jürgen Floege

**Affiliations:** Division of Nephrology and Clinical Immunology, RWTH University of Aachen, Pauwelsstr. 30, 52057 Aachen, Germany

**Keywords:** Magnesium, Chronic kidney disease, Cardiovascular disease, Diabetes, Depression

## Abstract

Magnesium fulfils important roles in multiple physiological processes. Accordingly, a tight regulation of magnesium homeostasis is essential. Dysregulated magnesium serum levels, in particular hypomagnesaemia, are common in patients with chronic kidney disease (CKD) and have been associated with poor clinical outcomes. In cell culture studies as well as in clinical situations magnesium levels were associated with vascular calcification, cardiovascular disease and altered bone-mineral metabolism. Magnesium has also been linked to diseases such as metabolic syndrome, diabetes, hypertension, fatigue and depression, all of which are common in CKD. The present review summarizes and discusses the latest clinical data on the impact of magnesium and possible effects of higher levels on the health status of patients with CKD, including an outlook on the use of magnesium-based phosphate-binding agents in this context.

## Introduction

**Fig. 1:**
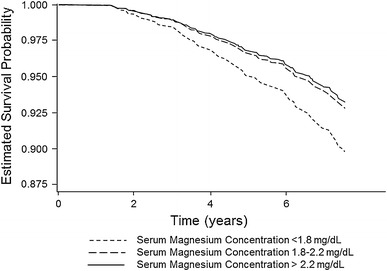
Estimated survival probabilities in CKD patients with high (>2.2 mg/dL/>0.90 mmol/L), medium (1.8–2.2 mg/dL/0.74–0.90 mmol/L) and low (<1.8 mg/dL/<0.74 mmol/L) serum magnesium concentrations (adapted and reprinted from [2] with permission from Elsevier)

As the fourth most abundant cation in the body, magnesium fulfils an important role in multiple physiological processes. Over 300 enzymes require the presence of magnesium for their catalytic action, including many enzymes utilising or synthesising ATP, or those that use other nucleotides to synthesise DNA and RNA. While clinical issues regarding magnesium disorders had received surprisingly little attention until the 1990s, a shift in focus led to some clinical investigations, especially in patients with chronic kidney disease (CKD). In 2012, several reviews on magnesium metabolism and disorders in magnesium balance were published in a special issue of the *Clinical Kidney Journal* [1]. Given the growing interest in the molecule since then the literature on the role of magnesium in CKD has continued to accumulate substantially. For instance, two cohort studies established hypomagnesaemia as a predictor of mortality (Fig. 1) and kidney function decline in CKD patients [2] as well as mortality in haemodialysis (HD) patients [3]. Furthermore, magnesium was identified as an independent risk factor for non-recovery of renal function in a cohort of critically ill patients with acute kidney injury [4].

This review examines and reviews the clinical impact of magnesium on the health status of CKD patients, in particular taking into account the influence of magnesium on diseases such as metabolic syndrome, diabetes, hypertension, vascular calcification and cardiovascular events, fatigue and depression all of which are frequently present in CKD patients and/or contribute to CKD progress.

## Magnesium and vascular calcification

Cardiovascular disease (CVD) is the leading cause of death in the CKD population [5]. The high prevalence of vascular calcification contributes significantly to this cardiovascular risk [6]. The molecular mechanisms leading to vascular calcification in CKD patients are still under investigation, but there is consensus that it is an active, multifactorial, cell-mediated and dynamic process [7, 8]. The progressive loss of kidney function is accompanied by elevated serum fibroblast growth factor 23 (FGF23) levels, a decrease in inorganic phosphate excretion and dysregulation of mineral and bone metabolism. These disturbances promote vascular calcification, whereby vascular smooth muscle cells (VSMCs) play a central role in the pathogenesis by undergoing an osteochondrogenic phenotype change in response to elevated phosphate levels [9].

Several cell culture and animal studies suggest a protective role of magnesium through multiple molecular mechanisms [10–12]. These results were extended by recent studies in which magnesium was shown to inhibit phosphate-induced calcification in vitro [13]. Moreover, higher magnesium levels prevented calcification of bovine VSMCs, inhibited expression of osteogenic proteins, apoptosis and further progression of already established calcification [14]. The first in vitro evidence in human aortic VSMCs for a protective role of magnesium on phosphate-induced calcification was based on the observation that living cells are necessary for magnesium ions to exert its protective effect. These studies suggested a potentially active intracellular role for magnesium ions in attenuating the vascular calcification process [15]. Additionally, increasing magnesium concentrations improved cell viability and normalised the cellular release of proteins involved in vascular calcification [15]. Inhibition of the Wnt/β-catenin signalling pathway was identified as one of the intracellular mechanisms by which the anti-calcifying effect of magnesium is achieved [16]. In the context of these data, microcalcifications in human atherosclerotic lesions contain both calcium and magnesium (in the form of whitlockite and calcium phosphate/apatite); whereas CKD- accelerated calcification was associated with a predominant deposition of calcium phosphate/apatite [17]. This suggests that in CKD, local magnesium homoeostasis is disturbed and potentially aggravates vascular calcifications.

The clinical relation between serum magnesium and vascular changes including calcification was assessed in several recent studies. A prospective study in 47 HD patients revealed an association of magnesium serum concentration with the intima–media thickness of carotid arteries [13]. In addition, CKD patients with higher magnesium serum concentrations had a significantly lower pulse wave velocity (PWV). Similarly, a cohort study of 512 renal transplant recipients identified low magnesium levels as a predictor of PWV and thus of vascular stiffness, independent of clinically relevant covariates and especially in older patients [18]. Furthermore, an observational cohort study, investigating 283 CKD patients, reported an association of high magnesium levels with less endothelial dysfunction [19].

So far, one double-blind, placebo-controlled randomised trial examined the efficacy of oral magnesium oxide (440 mg three times per week for 6 months) on endothelial function in HD patients [20]. While magnesium supplementation significantly decreased carotid intima–media thickness, there were no significant effects on C-reactive protein or flow-mediated dilatation, i.e. a functional endothelial marker [20]. The study was limited by the small sample size (less than 30 patients per treatment arm) and a significant baseline imbalance in intima–media thickness between the groups. Another small study in 47 dialysis patients randomised to no therapy or 610 mg magnesium citrate orally every other day for 2 months also noted a reduction of carotid intima–media thickness accompanied by a reduction in PTH levels [21]. Thus, more prospective interventional studies are warranted to assess the potential benefits of magnesium supplementation on vascular dysfunction and calcification.

## Magnesium and CVD

A meta-analysis of 19 prospective studies including 532,979 participants found a significant inverse association between magnesium intake and/or serum levels and the risk of CVD events in different patient populations [22]. This relationship was further sustained by a comprehensive study in 7,216 Spanish high-risk patients for cardiovascular disease. Again, an inverse association was noted between dietary magnesium intake and all-cause mortality [23].

While the above studies did not focus on CKD patients, several smaller studies did. In 80 diabetic CKD stage 2–4 patients, low serum magnesium was identified as a significant risk factor for an elevated pulse pressure, an established marker for cardiovascular mortality [24]. Analyses in 191 diabetics with CKD stage 1–3 have further underlined the relevance of this finding as lower magnesium levels are associated with increased mortality and accelerated progression of renal disease [25]. A third observational cohort study in 283 CKD patients also identified magnesium as an independent predictor of future cardiovascular outcomes [19]. In this study, Kaplan–Meier curves showed significantly higher cardiovascular mortality rates in CKD patients whose serum magnesium levels were below 2.05 mg/dL (0.84 mmol/L). Finally, these results were confirmed in a recent registry-based cohort study of 142,555 HD patients that again identified low serum magnesium as a significant predictor of cardiovascular mortality [3].

Thus, observational studies consistently identify low serum magnesium levels as a predictor or risk factor of vascular pathology, cardiovascular morbidity and all-cause mortality. However, recent large-scale randomised clinical trials [the Fourth International Study of Infarct Survival (ISIS 4) and Magnesium in Coronaries (MAGIC)] in non-renal patients could not prove a benefit of intravenous magnesium after myocardial infarction, and magnesium therapy is presently only indicated in patients with life-threatening ventricular arrhythmias [26]. Whether the situation is different in CKD patients with ischaemic heart disease remains to be tested.

## Magnesium and bone-mineral metabolism

The association between magnesium and mineral metabolism was recently investigated in several animal studies. To elucidate the effect of magnesium on phosphate homoeostasis, rats were fed either a normal or magnesium-deficient diet. Magnesium deficiency induced high serum FGF23 levels, possibly contributing to the observed decrease in renal phosphorus reabsorption [27]. While direct effects of magnesium on FGF-23 are not well established, the administration of a calcium acetate/magnesium carbonate (CaMg) containing phosphate binder in dialysis patients lowered both serum phosphate and FGF-23 [28]; in another study magnesium oxide also lowered FGF-23 levels in dialysis patients [29]; high magnesium concentrations can also activate the calcium-sensing receptor and thereby modulate PTH secretion in similar manner to calcium, albeit less potently [30, 31]. Another study demonstrated increasing serum 1,25-dihydroxyvitamin D and decreasing PTH as well as phosphate levels in response to magnesium loading in mouse models with genetic inactivation of PTH or both, PTH and the calcium-sensing receptor [32]. Both studies indicate the importance of balanced magnesium intake in terms of regulated phosphorus levels.

Since clinical studies have demonstrated the efficacy of magnesium-containing phosphate binders [33], and in line with the study by Quinn and colleagues [32], some concern has arisen that high serum magnesium or magnesium loading might oversuppress PTH secretion. In this context, an in vitro study with intact rat parathyroid glands is of importance, which showed that parathyroid glands were sensitive to an inhibitory effect of magnesium only when a moderately low calcium concentration was present [34]. The general dialysis population, however, does not have low calcium concentrations; and this issue warrants more clinical studies.

In addition to influencing PTH secretion, magnesium affects the synthesis and metabolism of vitamin D [32]. Many epidemiologic studies suggest that low vitamin D status may be associated with an increased risk of all-cause mortality [35–37]. Importantly, the activities of three major enzymes determining 25-hydroxyvitamin D_3_ (25(OH)D_3_) level [38–41] and vitamin D-binding protein [39] are magnesium dependent. Indeed, a cohort study including 12 157 NHANES III participants indicated that the inverse associations between serum 25(OH)D_3_ and risk of mortality could be modified by the intake level of magnesium [42].

Although once again randomised controlled studies are missing, the importance of magnesium for the mineral metabolism seems likely.

Effects of magnesium on bone in uremic patients have been reviewed recently [43]. In summary, various authors have hypothesized that magnesium might contribute to osteomalacia and/or renal osteodystrophy in particular via suppressing PTH [43]. However, in vivo confirmation of this in dialysis patients is lacking and there is at present no conclusive evidence that magnesium administration in CKD is associated with adynamic bone disease. Of note, in the CALMAG trial, administration of a CaMg-containing phosphate binder to dialysis patients did not affect markers of bone turnover over 6 months [28]. Epidemiological studies in non-CKD populations have linked magnesium deficiency to low bone mass and osteoporosis [44].

## Magnesium and hypertension

Previous studies on the risk of hypertension and ischaemic heart disease have shown only a modest effect [45–48], or inconsistent results [49–54], regarding its correlation to dietary magnesium intake (and serum magnesium levels). This was mainly due to a lack of direct measures of actual magnesium uptake. Serum levels of magnesium only correlate weakly with its intake unless extreme conditions prevail (e.g. excessive oral administration of magnesium salts) [55]. Thus, the use of urinary magnesium excretion as a more precise indicator of dietary magnesium uptake might provide a better insight into this association. In fact, results from a recent prospective population-based cohort study with 5,511 participants free of hypertension at baseline, demonstrated that urinary magnesium excretion was inversely associated with the risk of developing hypertension [56]. The same study group investigated the association between magnesium uptake and ischaemic heart disease in 7,664 participants free from known CVD at baseline. Again, low urinary magnesium excretion was independently associated with a higher incidence risk [57]. The authors suggest that an increased dietary magnesium intake, in particular in those persons with a low urinary excretion of magnesium, could reduce the risk of ischaemic heart disease, lower blood pressure and prevent hypertension [56, 57]. A recently published cross-sectional study, involving 175 healthy subjects, supports the proposed pathophysiological role of magnesium in the development of hypertension: here a lower magnesium concentration was the only significant parameter in a multivariate analysis between pre-hypertensive and normotensive subjects [58]. However, so far clinical trials have not detected significant antihypertensive effects of magnesium supplementation alone [59] but rather suggest that magnesium might augment the response to antihypertensive drugs [60].

## Magnesium and diabetes mellitus

**Fig. 2:**
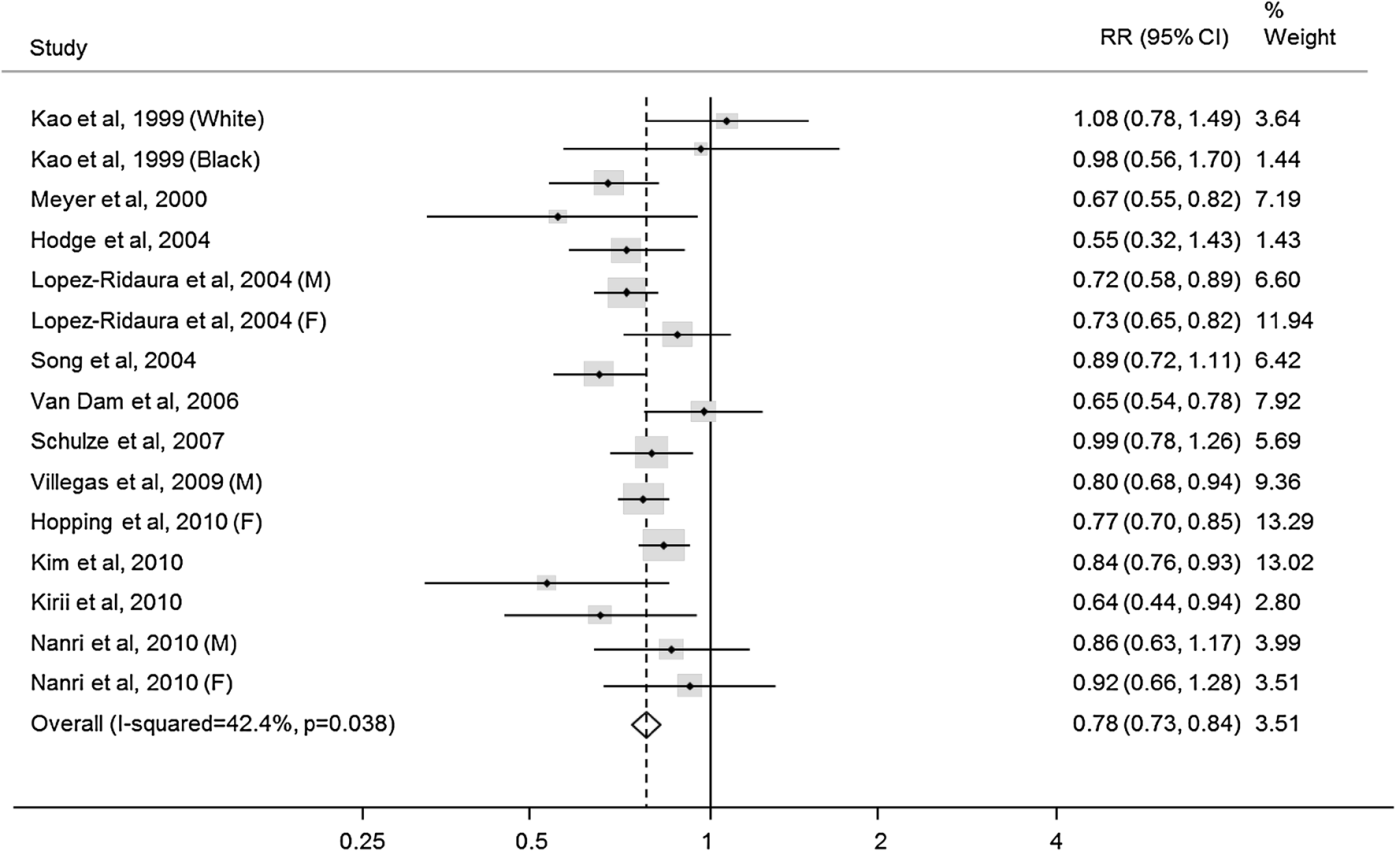
Meta-analysis of prospective cohort studies examining magnesium intake and the risk of developing type 2 diabetes. *M* male, *F* female (adapted and reprinted from [63]. Copyright and all rights reserved. Material from this publication has been used with the permission of American Diabetes Association)

Diabetes is the most frequent primary cause of CKD [61]. Hypomagnesaemia occurs with an incidence of 14–48 % among patients with T2DM compared with 3–15 % among their counterparts without diabetes [62]. A recent meta-analysis of thirteen prospective cohort studies involving 536,318 participants and 24,516 cases of diabetes provided further evidence that magnesium intake is inversely associated with the risk of T2DM [relative risk (RR) 0.78 (95 % CI 0.73–0.84)] in a dose–response manner (Fig. 2) [63]. Magnesium deficiency has also been linked to the development of the disease as well as its severity: the lower the magnesium level, the faster the deterioration of renal function in patients with type 2 diabetes mellitus (T2DM) [64].

Correction of hypomagnesaemia via dietary magnesium supplementation improved glucose handling and insulin response in elderly and non-insulin-dependent diabetics [65] and improved insulin sensitivity as well as metabolic control in T2DM patients with decreased serum magnesium levels [66]. The relationship between magnesium intake and metabolic parameters was further investigated in a cross-sectional study involving 210 elderly T2DM patients, of which 89 % exhibited a low magnesium intake and 37 % had overt hypomagnesaemia [67]. Metabolic syndrome and depression were associated with low intake, but not metabolic parameters, such as HbA1c, low high-density cholesterol, triglycerides and blood pressure.

In support of the well-known association between lower serum magnesium levels and impaired renal function, results from a cross-sectional study involving 51 T2DM patients showed that CKD was accompanied by hypomagnesaemia including low intracellular magnesium content of red cells most likely as a result of low intake [68]. In this study, magnesium deficiency was associated with poor blood glucose control and thus a potential increased risk of subsequent CVD events.

In line with this, T2DM is known to significantly increase the risk of ventricular arrhythmias, which represent a serious issue in CKD patients [69]. Results from a recent cross-sectional health survey among 750 adults with high T2DM prevalence showed that in diabetics the odds ratio of premature ventricular complexes was 0.24 (95 % CI 0.06–0.98) if serum magnesium was above 0.70 mmol/L compared to those where it was ≤0.70 mmol/L [70]. Thus, subnormal serum magnesium may be a contributor to arrhythmias among patients with T2DM, and conceivably magnesium supplementation in adults with T2DM may confer protection against ventricular arrhythmias.

Despite the growing body of evidence on the relation between hypomagnesaemia and insulin resistance in T2DM, the molecular aetiology is poorly understood. Transient receptor potential membrane melastatin (TRPM)-6 is an ion channel which is crucial for magnesium homoeostasis and plays an essential role in epithelial magnesium transport as well as in the active magnesium reabsorption in the gut and kidney. Two rare single nucleotide polymorphisms in TRPM6 (V1393I, K1584E) conferred susceptibility for T2DM but only if magnesium intake was low (<250 mg/day) [71]. Insulin stimulates TRPM6 activity via elevating the cell surface expression of TRPM6 but this mechanism fails with the above genetic variants TRPM6 [72]. Thus, these studies identify a direct molecular link between diabetes and magnesium and thus potentially link magnesium homoeostasis to diabetic outcomes.

Serum magnesium levels negatively correlated with HbA1c, fasting plasma glucose and microalbuminuria [73]. To better elucidate the relationship between magnesium deficiency and advanced T2DM nephropathy, a retrospective cohort study was conducted in 455 CKD patients. Hypomagnesaemia was significantly associated with and independently predicted progression to ESRD in patients with T2DM nephropathy but not in those with non-diabetic CKD [74]. In line with this observation, a later publication linked magnesium concentrations to the rate of kidney function decline [2]. However, in this latter study, the effect of magnesium lost significance after adjustment for additional covariates, in particular diuretics. Thus, whether magnesium supplementation may delay the onset of T2DM or its renal complications remains unknown at present.

## Magnesium and fatigue/depression

Fatigue involves general, mental and physical fatigue reduced motivation and reduced activity dimensions [75]. It is one of the most frequent dialysis-associated symptoms with prevalences reported from 60 and up to 97 % [76]. Fatigue is often accompanied by depression, and major depressive disorders are as well very common among CKD and ESRD patients, affecting 20 % of CKD patients compared to a prevalence of 2–10 % in the general population [77]. The fact that only a minority of affected CKD patients (~20 %) receives an adequate diagnosis and treatment for depression, poses a major challenge for clinicians to develop strategies to better understand and manage depression in this population [77].

Basic research reveals the involvement of magnesium ions in pathways which are connected to the known pathophysiology of depression. The *N*-methyl-d-aspartate (NMDA)-ergic system received marked attention in the context of developing new compounds for mood disorders in recent years [78]. The magnesium ion is a naturally occurring NMDA-receptor antagonist, and reduced intracellular magnesium ions can be responsible for an increased NMDA receptor sensitivity [79]. Besides NMDA targets involved in the antidepressant action of magnesium, there are brain-derived neurotrophic factors (BDNF) and glycogen synthase kinase-3 (GSK-3). Magnesium increases BDNF and both inhibit the activity of GSK-3, an enzyme involved in the mechanisms of action of antidepressants [80]. Magnesium is also a cofactor of tryptophan hydroxylase, which catalyses serotonin synthesis. Hence levels of serotonin and BDNF increase in the presence of magnesium, leading to an improvement of depressive symptoms.

Animal data indicated that short-term administration of magnesium possesses potent antidepressant-like properties in the forced swim test in mice [81]. No development of tolerance was observed after prolonged treatment [82].

An early study in non-renal patients with chronic fatigue indeed noted low intracellular magnesium levels, and magnesium supplementation led to some clinical improvement [83]. Further evidence was gained in one randomised controlled trial comparing the efficacy of oral magnesium supplementation with the standard antidepressant imipramine in a 12-week treatment of newly diagnosed depression in twenty-three elderly with T2DM and hypomagnesaemia. Here, oral magnesium supplementation with MgCl_2_ (equivalent to 450 mg of elemental magnesium) was as effective as 50 mg imipramine daily, the prototypic medication in depression [84]. However, of note, no magnesium study has so far specifically targeted CKD-associated fatigue and depression. Given the paucity of the safety data of currently available antidepressants in CKD patients, magnesium supplementation—provided that serum magnesium levels are controlled—could become a safe therapeutic option in this population.

## Conclusion

The clinical role of magnesium in terms of benefits and harms of higher or lower serum magnesium levels continues to attract growing attention in research as evidenced by the increasing literature available on this topic. In CKD patients, vascular calcification, hypertension, diabetes, and diabetic nephropathy are common comorbid situations associated with increased mortality. All of these factors are potentially affected by magnesium, and there is accumulating evidence for beneficial effects of magnesium supplementation and slightly elevated magnesium levels. However, it is important to stress that in most studies discussed above, causality cannot be inferred from the associations reported. For example, many of the associations between magnesium intake and outcome may represent true relationships but also could be confounded by magnesium intake reflecting different general dietary and/or life-style habits. As such, we need intervention studies to confirm or refute the hypotheses derived from these associations.

**Fig. 3:**
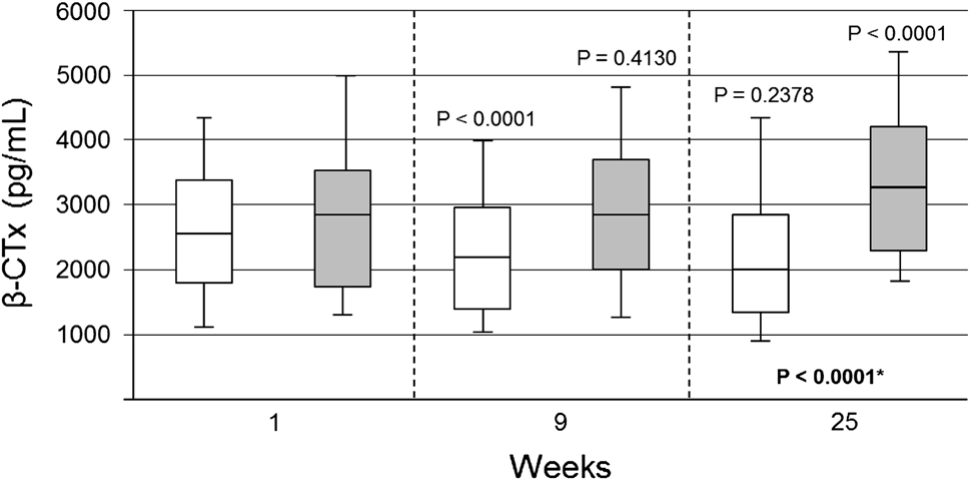
Impact of phosphate binders on serum levels of the bone turnover marker β-CTX (beta-crosslaps). Time course of values at weeks 9 and 25 of the calcium acetate/magnesium carbonate group (*n* = 105) and of the sevelamer-HCl group (*n* = 99) is displayed in *white* and *grey*, respectively (adapted and reprinted from [28] by permission of Oxford University Press)

In particular indications, such intervention studies already exist. Thus, in hyperphosphataemia associated with advanced CKD, phosphate binders are often necessary to limit dietary phosphate absorption. Here, the combination of calcium acetate and magnesium carbonate has been shown to be as efficient and equally well tolerated as sevelamer hydrochloride [33] in addition to reducing calcium intake compared to pure calcium-based phosphate binders [85]. In an animal study, the ensuing mildly elevated magnesium levels resulted in beneficial effects in terms of calcification, PTH levels and survival [86]. Clinical data further demonstrated that CaMg does not negatively influence bone health and might even help to maintain it, causing neither an overstimulation nor a suppression of bone turnover (Fig. 3) [28]. In view of the above discussion, therapy with CaMg in hyperphosphataemic CKD patients offers the exciting option to also evaluate whether the many pleiotropic actions of magnesium beneficially influence health issues beyond bone mineral disease.

## References

[1] Luft C (2012) Magnesium—a versatile and often overlooked element: new perspectives with a focus on chronic kidney disease. Clin Kidney J 5(Suppl 1). doi:10.1093/ndtplus/sfs035 10.1093/ndtplus/sfs035 PMC4455823 26069823

[2] Van Laecke S, Nagler EV, Verbeke F, Van Biesen W, Vanholder R (2013). Hypomagnesemia and the risk of death and GFR decline in chronic kidney disease. Am J Med.

[3] Sakaguchi Y, Fujii N, Shoji T, Hayashi T, Rakugi H, Isaka Y (2014). Hypomagnesemia is a significant predictor of cardiovascular and non-cardiovascular mortality in patients undergoing hemodialysis. Kidney Int.

[4] Alves SC, Tomasi CD, Constantino L (2013). Hypomagnesemia as a risk factor for the non-recovery of the renal function in critically ill patients with acute kidney injury. Nephrol Dial Transplant.

[5] Foley RN, Parfrey PS (1998). Cardiovascular disease and mortality in ESRD. J Nephrol.

[6] Shanahan CM (2006). Vascular calcification—a matter of damage limitation?. Nephrol Dial Transplant.

[7] London GM (2013). Mechanisms of arterial calcifications and consequences for cardiovascular function. Kidney Int Suppl.

[8] Massy ZA, Drüeke TB (2012). Magnesium and outcomes in patients with chronic kidney disease: focus on vascular calcification, atherosclerosis and survival. Clin Kidney J.

[9] Tyson KL, Reynolds JL, McNair R, Zhang Q, Weissberg PL, Shanahan CM (2003). Osteo/chondrocytic transcription factors and their target genes exhibit distinct patterns of expression in human arterial calcification. Arterioscler Thromb Vasc Biol.

[10] Peters F, Epple M (2001). Simulating arterial wall calcification in vitro: biomimetic crystallization of calcium phosphates under controlled conditions. Z Kardiol.

[11] Altura BM, Altura BT, Carella A, Gebrewold A, Murakawa T, Nishio A (1987). Mg^2+^–Ca^2+^ interaction in contractility of vascular smooth muscle: Mg^2+^ versus organic calcium channel blockers on myogenic tone and agonist-induced responsiveness of blood vessels. Can J Physiol Pharmacol.

[12] Ivanovski O, Szumilak D, Nguyen-Khoa T, Ruellan N, Phan O, Lacour B, Descamps-Latscha B, Drüeke TB, Massy ZA (2005). The antioxidant N-acetylcysteine prevents accelerated atherosclerosis in uremic apolipoprotein E knockout mice. Kidney Int.

[13] Salem S, Bruck H, Bahlmann FH (2012). Relationship between magnesium and clinical biomarkers on inhibition of vascular calcification. Am J Nephrol.

[14] Kircelli F, Peter ME, Sevinc Ok E, Celenk FG, Yilmaz M, Steppan S, Asci G, Ok E, Passlick-Deetjen J (2012). Magnesium reduces calcification in bovine vascular smooth muscle cells in a dose-dependent manner. Nephrol Dial Transplant.

[15] Louvet L, Büchel J, Steppan S, Passlick-Deetjen J, Massy ZA (2013). Magnesium prevents phosphate-induced calcification in human aortic vascular smooth muscle cells. Nephrol Dial Transplant.

[16] Montes de Oca A, Guerrero F, Martinez-Moreno JM (2014). Magnesium inhibits Wnt/β-catenin activity and reverses the osteogenic transformation of vascular smooth muscle cells. PLoS ONE.

[17] Fischer DC, Behetes GJ, Hakenberg OW, Voigt M, Vervaet BA, Robijn S, Kundt G, Schareck W, D’Haese PC, Haffner D (2012). Arterial microcalcification in atherosclerotic patients with and without chronic kidney disease: a comparative high-resolution scanning X-ray diffraction analysis. Calcif Tissue Int.

[18] Van Laecke S, Maréchal C, Verbeke F, Peeters P, Van Biesen W, Devuyst O, Jadoul M, Vanholder R (2011). The relation between hypomagnesaemia and vascular stiffness in renal transplant recipients. Nephrol Dial Transplant.

[19] Kanbay M, Yilmaz MI, Apetrii M (2012). Relationship between serum magnesium levels and cardiovascular events in chronic kidney disease patients. Am J Nephrol.

[20] Mortazavi M, Moeinzadeh F, Saadatnia M, Shahidi S, McGee JC, Minagar A (2013). Effect of magnesium supplementation on carotid intima–media thickness and flow-mediated dilatation among hemodialysis patients: a double-blind, randomized, placebo-controlled trial. Eur Neurol.

[21] Turgut F, Kanbay M, Metin M, Uz E, Akcay A, Covic A (2008). Magnesium supplementation helps to improve carotid intima media thickness in patients on hemodialysis. Int Urol Nephrol.

[22] Qu X, Jin F, Hao Y, Li H, Tang T, Wang H, Yan W, Dai K (2013). Magnesium and the risk of cardiovascular events: a meta-analysis of prospective cohort studies. PLoS ONE.

[23] Guasch-Ferré M, Bulló M, Estruch R (2014). Dietary magnesium intake is inversely associated with mortality in adults at high cardiovascular disease risk. J Nutr.

[24] Fragoso A, Silva AP, Gundlach K, Janine Büchel J, Leão Neves P (2014) Magnesium and FGF-23 are independent predictors of pulse pressure in pre-dialysis diabetic chronic kidney disease patients. Clin Kidney J 7(2):161–166. doi:10.1093/ckj/sfu003 10.1093/ckj/sfu003 PMC4377779 25852865

[25] Silva AP, Fragoso A, Silva C, Tavares N, Santos N, Martins H, Gundlach K, Büchel J, Camacho A, Faísca M, Jesus Varela I, Neves P (2014). Magnesium and mortality in patients with diabetes and early chronic kidney disease. J Diabetes Metab.

[26] Shechter M (2003). Does magnesium have a role in the treatment of patients with coronary artery disease?. Am J Cardiovasc Drugs.

[27] Matsuzaki H, Kajita Y, Miwa M (2013). Magnesium deficiency increases serum fibroblast growth factor-23 levels in rats. Magnes Res.

[28] Covic A, Passlick-Deetjen J, Kroczak M, Büschges-Seraphin B, Ghenu A, Ponce P, Marzell B, de Francisco AL (2013). A comparison of calcium acetate/magnesium carbonate and sevelamer-hydrochloride effects on fibroblast growth factor-23 and bone markers: post hoc evaluation from a controlled, randomized study. Nephrol Dial Transplant.

[29] Iguchi A, Watanabe Y, Iino N, Kazama JJ, Iesato H, Narita I (2014) Serum magnesium concentration is inversely associated with fibroblast growth factor 23 in haemodialysis patients. Nephrology. 10.1111/nep.12287 10.1111/nep.12287 24899171

[30] Brown EM (1991). Extracellular Ca2+ sensing, regulation of parathyroid cell function, and role of Ca2+ and other ions as extracellular (first) messengers. Physiol Rev.

[31] Kumar R, Thompson JR (2011). The regulation of parathyroid hormone secretion and synthesis. J Am Soc Nephrol.

[32] Quinn SJ, Thomsen AR, Egbuna O, Pang J, Baxi K, Goltzman D, Pollak M, Brown EM (2013). CaSR-mediated interactions between calcium and magnesium homeostasis in mice. Am J Physiol Endocrinol Metab.

[33] de Francisco AL, Leidig M, Covic AC, Ketteler M, Benedyk-Lorens E, Mircescu GM, Scholz C, Ponce P, Passlick-Deetjen J (2010). Evaluation of calcium acetate/magnesium carbonate as a phosphate binder compared with sevelamer hydrochloride in haemodialysis patients: a controlled randomized study (CALMAG study) assessing efficacy and tolerability. Nephrol Dial Transplant.

[34] Rodríguez-Ortiz ME, Canalejo A, Herencia C (2014). Magnesium modulates parathyroid hormone secretion and upregulates parathyroid receptor expressions at moderately low calcium concentration. Nephrol Dial Transplant.

[35] Holick MF (2006). Resurrection of vitamin D deficiency and rickets. J Clin Invest.

[36] Zittermann A, Iodice S, Pilz S, Grant WB, Bagnardi V, Gandini S (2012). Vitamin D deficiency and mortality risk in the general population: a meta-analysis of prospective cohort studies. Am J Clin Nutr.

[37] Schöttker B, Ball D, Gellert C, Brenner H (2013). Serum 25-hydroxyvitamin D levels and overall mortality. A systematic review and meta-analysis of prospective cohort studies. Ageing Res Rev.

[38] Risco F, Traba ML (1992). Influence of magnesium on the in vitro synthesis of 24,25-dihydroxyvitamin D3 and 1 alpha, 25-dihydroxyvitamin D3. Magnes Res.

[39] Rude RK, Adams JS, Ryzen E, Endres DB, Niimi H, Horst RL, Haddad JG, Singer FR (1985). Low serum concentrations of 1,25-dihydroxyvitamin D in human magnesium deficiency. J Clin Endocrinol Metab.

[40] Risco F, Traba ML (1994). Possible involvement of a magnesium dependent mitochondrial alkaline phosphatase in the regulation of the 25-hydroxyvitamin D3-1 alpha-and 25-hydroxyvitamin D3-24R-hydroxylases in LLC-PK1 cells. Magnes Res.

[41] Rösler A, Rabinowitz D (1973). Magnesium-induced reversal of vitamin-D resistance in hypoparathyroidism. Lancet.

[42] Deng X, Song Y, Manson JE, Signorello LB, Zhang SM, Shrubsole MJ, Ness RM, Seidner DL, Dai Q (2013). Magnesium, vitamin D status and mortality: results from US National Health and Nutrition Examination Survey (NHANES) 2001 to 2006 and NHANES III. BMC Med.

[43] Cunningham J, Rodríguez M, Messa P (2012). Magnesium in chronic kidney disease stages 3 and 4 and in dialysis patients. Clin Kidney J.

[44] Rude RK, Singer FR, Gruber HE (2009). Skeletal and hormonal effects of magnesium deficiency. J Am Coll Nutr.

[45] Witteman JC, Willett WC, Stampfer MJ, Colditz GA, Sacks FM, Speizer FE, Rosner B, Hennekens CH (1989). A prospective study of nutritional factors and hypertension among US women. Circulation.

[46] Ascherio A, Rimm EB, Giovannucci EL, Colditz GA, Rosner B, Willett WC, Sacks F, Stampfer MJ (1992). A prospective study of nutritional factors and hypertension among US men. Circulation.

[47] Ascherio A, Hennekens C, Willett WC, Sacks F, Rosner B, Manson J, Witteman J, Stampfer MJ (1996). Prospective study of nutritional factors, blood pressure, and hypertension among US women. Hypertension.

[48] Song Y, Sesso HD, Manson JE, Cook NR, Buring JE, Liu S (2006). Dietary magnesium intake and risk of incident hypertension among middle-aged and older US women in a 10-year follow-up study. Am J Cardiol.

[49] Elwood PC, Fehily AM, Ising H, Poor DJ, Pickering J, Kamel F (1996). Dietary magnesium does not predict ischaemic heart disease in the Caerphilly cohort. Eur J Clin Nutr.

[50] Song Y, Manson JE, Cook NR, Albert CM, Buring JE, Liu S (2005). Dietary magnesium intake and risk of cardiovascular disease among women. Am J Cardiol.

[51] Al-Delaimy WK, Rimm EB, Willett WC, Stampfer MJ, Hu FB (2004). Magnesium intake and risk of coronary heart disease among men. J Am Coll Nutr.

[52] Liao F, Folsom AR, Brancati FL (1998). Is low magnesium concentration a risk factor for coronary heart disease? The Atherosclerosis Risk in Communities (ARIC) Study. Am Heart J.

[53] Ford ES (1999). Serum magnesium and ischaemic heart disease: findings from a national sample of US adults. Int J Epidemiol.

[54] Abbott RD, Ando F, Masaki KH, Tung KH, Rodriguez BL, Petrovitch H, Yano K, Curb JD (2003). Dietary magnesium intake and the future risk of coronary heart disease (the Honolulu Heart Program). Am J Cardiol.

[55] Xing JH, Soffer EE (2001). Adverse effects of laxatives. Dis Colon Rectum.

[56] Joosten MM, Gansevoort RT, Mukamal KJ, Kootstra-Ros JE, Feskens EJ, Geleijnse JM, Navis G, Bakker SJ, PREVEND Study Group (2013). Urinary magnesium excretion and risk of hypertension: the prevention of renal and vascular end-stage disease study. Hypertension.

[57] Joosten MM, Gansevoort RT, Mukamal KJ, van der Harst P, Geleijnse JM, Feskens EJ, Navis G, Bakker SJ, PREVEND Study Group (2013). Urinary and plasma magnesium and risk of ischemic heart disease. Am J Clin Nutr.

[58] Rodríguez-Moran M, Guerrero-Romero F (2014). Hypomagnesemia and prehypertension in otherwise healthy individuals. Eur J Intern Med.

[59] Dickinson HO, Nicolson DJ, Campbell F, Cook JV, Beyer FR, Ford GA, Mason J (2006). Magnesium supplementation for the management of primary hypertension in adults (Review). Cochrane Database Syst Rev.

[60] Rosanoff A (2010). Significant first presence of Mg symposium at experimental biology, a large USA conference. Magnes Res.

[61] ERA-EDTA Registry (2012) ERA-EDTA Registry Annual Report 2010. Academic Medical Center, Department of Medical Informatics, Amsterdam

[62] Pham PC, Pham PM, Pham SV, Miller JM, Pham PT (2007). Hypomagnesemia in patients with type 2 diabetes. Clin J Am Soc Nephrol.

[63] Dong JY, Xun P, He K, Qin LQ (2011). Magnesium intake and risk of type 2 diabetes: meta-analysis of prospective cohort studies. Diabetes Care.

[64] Pham PC, Pham PM, Pham PA, Pham SV, Pham HV, Miller JM, Yanagawa N, Pham PT (2005). Lower serum magnesium levels are associated with more rapid decline of renal function in patients with diabetes mellitus type 2. Clin Nephrol.

[65] Paolisso G, Sgambato S, Pizza G, Passariello N, Varricchio M, D’Onofrio F (1989). Improved insulin response and action by chronic magnesium administration in aged NIDDM subjects. Diabetes Care.

[66] Rodríguez-Morán M, Guerrero-Romero F (2003). Oral magnesium supplementation improves insulin sensitivity and metabolic control in type 2 diabetic subjects: a randomized double-blind controlled trial. Diabetes Care.

[67] Huang JH, Lu YF, Cheng FC, Lee JN, Tsai LC (2012). Correlation of magnesium intake with metabolic parameters, depression and physical activity in elderly type 2 diabetes patients: a cross-sectional study. Nutr J.

[68] Sales CH, Pedrosa LF, Lima JG, Lemos TM, Colli C (2011). Influence of magnesium status and magnesium intake on the blood glucose control in patients with type 2 diabetes. Clin Nutr.

[69] Escobedo LG, Caspersen CJ (1997). Risk factors for sudden coronary death in the United States. Epidemiology.

[70] Del Gobbo LC, Song Y, Poirier P, Dewailly E, Elin RJ, Egeland GM (2012). Low serum magnesium concentrations are associated with a high prevalence of premature ventricular complexes in obese adults with type 2 diabetes. Cardiovasc Diabetol.

[71] Song Y, Hsu YH, Niu T, Manson JE, Buring JE, Liu S (2009). Common genetic variants of the ion channel transient receptor potential membrane melastatin 6 and 7 (TRPM6 and TRPM7), magnesium intake, and risk of type 2 diabetes in women. BMC Med Genet.

[72] Nair AV, Hocher B, Verkaart S (2012). Loss of insulin-induced activation of TRPM6 magnesium channels results in impaired glucose tolerance during pregnancy. Proc Natl Acad Sci USA.

[73] Prabodh S, Prakash DS, Sudhakar G, Chowdary NV, Desai V, Shekhar R (2011). Status of copper and magnesium levels in diabetic nephropathy cases: a case–control study from South India. Biol Trace Elem Res.

[74] Sakaguchi Y, Shoji T, Hayashi T (2012). Hypomagnesemia in type 2 diabetic nephropathy: a novel predictor of end-stage renal disease. Diabetes Care.

[75] Bonner A, Wellard S, Caltabiano M (2008). Levels of fatigue in people with ESRD living in far North Queensland. Clin Nurs.

[76] Horigan A, Rocchiccioli J, Trimm D (2012). Dialysis and fatigue: implications for nurses—a case study analysis. Medsurg Nurs.

[77] Hedayati SS, Yalamanchili V, Finkelstein FO (2012). A practical approach to the treatment of depression in patients with chronic kidney disease and end-stage renal disease. Kidney Int.

[78] Li X, Frye MA, Shelton RC (2012). Review of pharmacological treatment in mood disorders and future directions for drug development. Neuropsychopharmacology.

[79] Murck H (2013). Ketamine, magnesium and major depression—from pharmacology to pathophysiology and back. J Psychiatr Res.

[80] Szewczyk B, Poleszak E, Sowa-Kućma M (2008). Antidepressant activity of zinc and magnesium in view of the current hypotheses of antidepressant action. Pharmacol Rep.

[81] Socała K, Nieoczym D, Poleszak E, Wlaź P (2012). Influence of the phosphodiesterase type 5 inhibitor, sildenafil, on antidepressant-like activity of magnesium in the forced swim test in mice. Pharmacol Rep.

[82] Poleszak E, Szewczyk B, Kedzierska E, Wlaź P, Pilc A, Nowak G (2004). Antidepressant- and anxiolytic-like activity of magnesium in mice. Pharmacol Biochem Behav.

[83] Cox IM, Campbell MJ, Dowson D (1991). Red blood cell magnesium and chronic fatigue syndrome. Lancet.

[84] Barragán-Rodríguez L, Rodríguez-Morán M, Guerrero-Romero F (2008). Efficacy and safety of oral magnesium supplementation in the treatment of depression in the elderly with type 2 diabetes: a randomized, equivalent trial. Magnes Res.

[85] Maduell F, Pérez N, Arias M (2012). Calcium acetate and magnesium carbonate association: a good alternative of calcium-based phosphate binders. Nephrol Dial Transplant.

[86] De Schutter TM, Behets GJ, Geryl H, Peter ME, Steppan S, Gundlach K, Passlick-Deetjen J, D’Haese PC, Neven E (2013). Effect of a magnesium-based phosphate binder on medial calcification in a rat model of uremia. Kidney Int.

